# Clinical-radiomics nomogram based on the fat-suppressed T2 sequence for differentiating luminal and non-luminal breast cancer

**DOI:** 10.3389/fonc.2024.1451414

**Published:** 2024-10-25

**Authors:** Yaxin Guo, Shunian Li, Jun Liao, Yuqi Guo, Yiyan Shang, Yunxia Wang, Qingxia Wu, Yaping Wu, Meiyun Wang, Hongna Tan

**Affiliations:** ^1^ Department of Radiology, People's Hospital of Zhengzhou University, Zhengzhou, Henan, China; ^2^ Department of Radiology, Henan Provincial People’s Hospital, Zhengzhou, Henan, China; ^3^ Department of Hepatobiliary and Pancreatic Surgery, People's Hospital of Zhengzhou University & Henan Provincial People’s Hospital, Zhengzhou, Henan, China; ^4^ Department of Radiology, People's Hospital of Henan University, Zhengzhou, Henan, China; ^5^ Beijing United Imaging Research Institute of Intelligent Imaging, United Imaging Intelligence Co., Ltd., Beijing, China

**Keywords:** breast cancer, MRI, radiomics, luminal breast cancer, peritumoral

## Abstract

**Objective:**

To establish and validate a new clinical-radiomics nomogram based on the fat-suppressed T2 sequence for differentiating luminal and non-luminal breast cancer.

**Methods:**

A total of 593 breast cancer patients who underwent preoperative breast MRI from Jan 2017 to Dec 2020 were enrolled, which were randomly divided into the training (n=474) and test sets (n=119) at the ratio of 8:2. Intratumoral region (ITR) of interest were manually delineated, and peritumoral regions of 3 mm and 5 mm (PTR-3 mm and PTR-5 mm) were automatically obtained by dilating the ITR. Intratumoral and peritumoral radiomics features were extracted from the fat-suppressed T2-weighted images, including first-order statistical features, shape features, texture features, and filtered features. The Mann-Whitney U Test, Z score normalization, K-best method, and least absolute shrinkage and selection operator (LASSO) algorithm were applied to select key features to construct radscores based on ITR, PTR-3 mm, PTR-5 mm, ITR+PTR-3 mm and ITR+ PTR-5 mm. Risk factors were selected by univariate and multivariate logistic regressions and were used to construct a clinical model and a clinical-radiomics model that presented as a nomogram. The performance of models was assessed by sensitivity, specificity, accuracy, the area under the curve (AUC) of receiver operating characteristic (ROC), calibration curves, and decision curve analysis (DCA).

**Results:**

ITR+PTR-3 mm radsore and histological grade were selected as risk factors. A clinical-radiomics model was constructed by adding ITR+PTR-3mm radscore to the clinical factor, which was presented as a nomogram. The clinical-radiomics nomogram showed the highest AUC (0.873), sensitivity (72.3%), specificity (78.9%) and accuracy (77.0%) in the training set and the highest AUC (0.851), sensitivity (71.4%), specificity (79.8%) and accuracy (77.3%) in the test set. DCA showed that the clinical-radiomics nomogram had the greatest net clinical benefit compared to the other models.

**Conclusion:**

The clinical-radiomics nomogram showed promising clinical application value in differentiating luminal and non-luminal breast cancer.

## 1 Introduction

Breast cancer is the most common cancer among women worldwide and seriously endangers women's physical and mental health (1, 2). Molecular subtypes play a crucial role in guiding clinical treatment decisions and assessing prognosis in breast cancer (3, 4). Breast cancer can be classified into luminal and non-luminal types according to hormone receptor status (5). Luminal cancers, which generally express estrogen and progesterone receptors, respond well to endocrine therapies and typically have a favorable prognosis (6, 7). In contrast, non-luminal cancers, including the human epidermal growth factor receptor2 (HER2)-overexpressing, and triple-negative types, do not respond to hormone therapies and generally have poorer outcomes (8). However, HER2-overexpressing cancers can have improved prognosis with targeted HER2 therapies. Additionally, non-luminal cancers show higher responsiveness to neoadjuvant therapies, achieving pathological complete response rates of 20-40% (9, 10). Therefore, accurate assessment of molecular subtypes of breast cancer before surgery is essential to develop personalized treatment strategies and improve prognosis. However, the primary method to distinguish luminal from non-luminal breast cancer, immunohistochemical analysis of core needle biopsy tissue, is invasive, and time-consuming (11). Furthermore, given the heterogeneity of breast cancer, a single biopsy sample may not be representative of the entire lesion (12–14). Therefore, there is a need for a noninvasive and efficient method to differentiate luminal and non-luminal molecular subtypes in breast cancer patients before surgery.

Although mammography, ultrasound, and MRI are common techniques used for the diagnosis and treatment evaluation of breast cancer, these traditional techniques cannot provide accurate evaluation for differentiating luminal and non-luminal molecular subtypes. Radiomics provides a new way for precision medicine and personalized treatment by extracting high-throughput image features that are invisible to the naked eye to comprehensively quantify tumor heterogeneity (15–17). It has been demonstrated that radiomics has significant clinical potential in breast cancer diagnosis, efficacy assessment, and prognosis prediction (18). Although dynamic contrast-enhanced (DCE)-MRI-based radiomics has been shown to differentiate molecular subtypes of breast cancer by capturing dynamic changes (19), T2-weighted imaging has high accuracy and sensitivity for assessing normal anatomical structures and identifying various pathological changes without the need for contrast agents, especially for patients with contraindications to contrast agents (20). Hence, our study aims to differentiate luminal and non-luminal breast cancer by establishing the radiomics features based on the fat-suppressed T2 sequence.

Previous studies mainly focused on the intratumoral region but ignored the peritumoral region. However, the microenvironment surrounding the tumor plays a crucial role in tumor growth, invasion, and metastasis (21, 22). Therefore, it is important to consider the microenvironment surrounding the tumor in the study of breast cancer. At present, there are still few studies published on the use of both intratumoral and peritumoral radiomics features for preoperative differentiating luminal and non-luminal molecular subtypes. The purpose of this study was to establish a new clinical-radiomics nomogram based on the fat-suppressed T2 sequence for preoperative differentiating luminal and non-luminal breast cancer, as well as to provide a reference for individualized treatment and prognostic assessment of breast cancer.

## 2 Methods

### 2.1 Patient population

**Figure 1:**
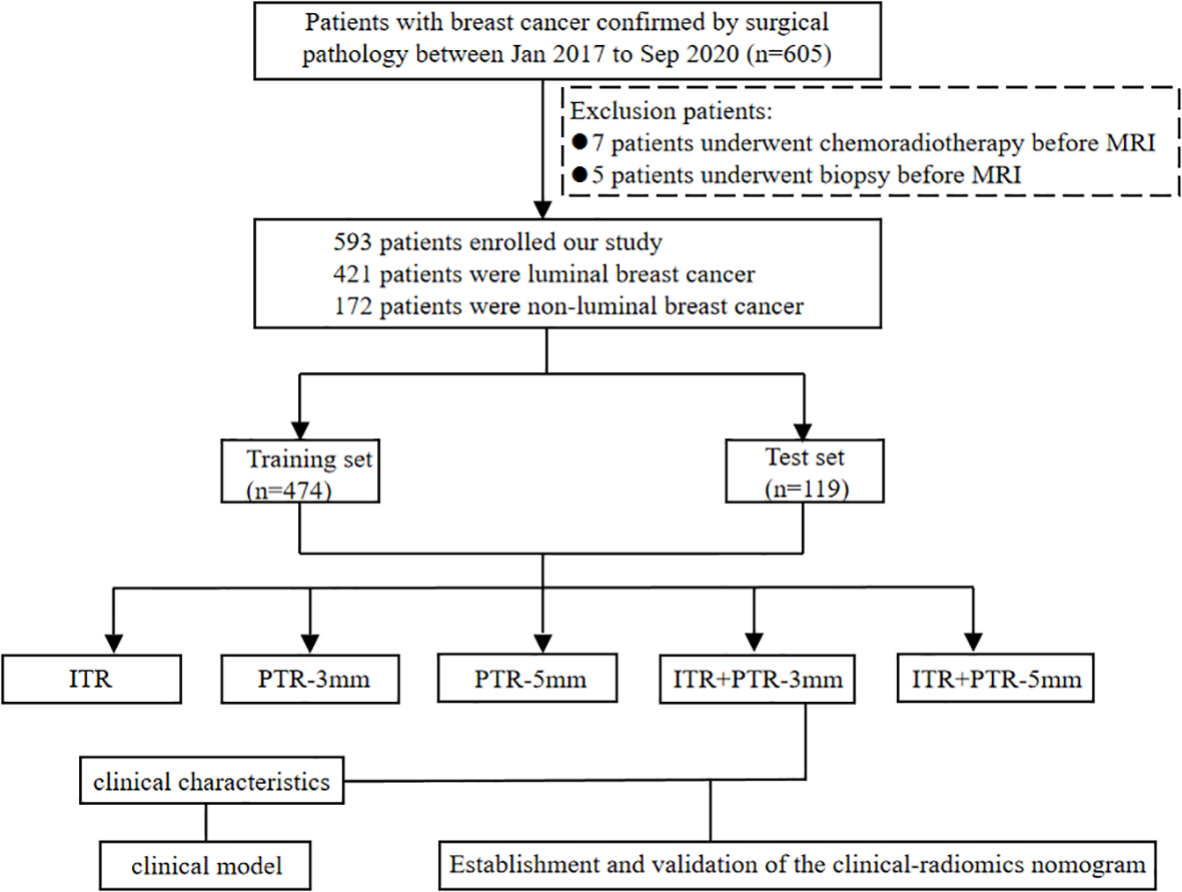
Flow chart for selection and grouping of populations according to inclusion and exclusion criteria.

The study was approved by the Ethics Committee of Henan Provincial People’s Hospital (No: 2022-124), and the participants informed consent requirement was waived. 605 patients who underwent initial MRI examinations in our hospital from Jan 2017 to Dec 2020 were retrospectively enrolled. Patients with definite pathological results were included in this study, while patients with missing data, or who underwent biopsy (5 patients), or chemoradiotherapy before MRI examination (7 patients) were excluded. In total, 593 patients were finally enrolled, including 421 patients with luminal type and 172 patients with non-luminal type. The luminal type included 108 cases of luminal A type and 313 cases of luminal B type; the non-luminal type included 103 cases of triple-negative type and 69 cases of HER2 over-expression type. The data of patients were divided into a training set (n=474) and a test set (n=119) at the ratio of 8:2. The flowchart of this study is shown in **Figure 1**.

### 2.2 Fat-Suppressed T2-weighted imaging acquisition

Breast MRI examination was performed by 3.0T MR imaging devices and dedicated breast phased-array surface coils (GE Medical Systems Discovery MR750, Milwaukee). The MRI protocol included an unenhanced T1-weighted sequence, a fat-suppressed T2-weighted sequence, and enhanced axial T1-weighted sequences. The main MRI sequence scanning parameters were as follows: T1-weighted FSE (fast spin-echo) sequence (TR/TE, 680/10ms; slice thickness, 5.0mm; field of view, 751×340mm; matrix scan, 512×512) and T2-weighted FSE-IDEAL ASSET (fast spin-echo with iterative dixon water-fat separation with echo asymmetry and least-squares estima and array spatial sensitivity encoding technique) sequence (TR/TE,4000/80ms; slice thickness, 5.0mm; field of view, 751×340mm; matrix scan, 512×512; NEX, 1), and DCE scanning was performed using T1-weighted VIBRANT(volume imaging for breast assessment) technique (TR/TE,3.8/1.6 ms; slice thickness, 1.1 mm; field of view, 751×340 mm; matrix scan, 512×512; phase, 8). The contrast medium (Gado-linium-DTPA; Magnevist, Schering, Germany, 0.2 mmol/kg) was intravenous administered as a bolus injection to the patients undergoing contrast-enhanced MRI, followed by a 20 ml saline flush.

### 2.3 Clinical characteristics

Clinical characteristics were obtained from the electronic medical records, including patient age, menstrual status, histological grade, and some radiological characteristics which included location, lesion size, background parenchymal enhancement (BPE), time-signal intensity curve (TIC), and MRI-reported axillary lymph node (ALN) status. Radiological characteristics were analyzed by 2 radiologists with more than 5 years of experience in breast imaging. They were blinded to the pathological results, and a consensus decision was made in cases of discrepancy. All the original images acquired after scanning were transmitted to the AW4.6 postprocessing workstation. Based on the second edition of the breast imaging reporting and data system (BI-RADS) for breast MRI, TIC was classified into 3 classical types: type I-inflow type, type II-platform type and type III-outflow type, and BPE was classified into 4 classical types: type I-minimal type, type II-mild type, type III-moderate type and type IV-marked type.

### 2.4 Image segmentation

**Figure 2:**
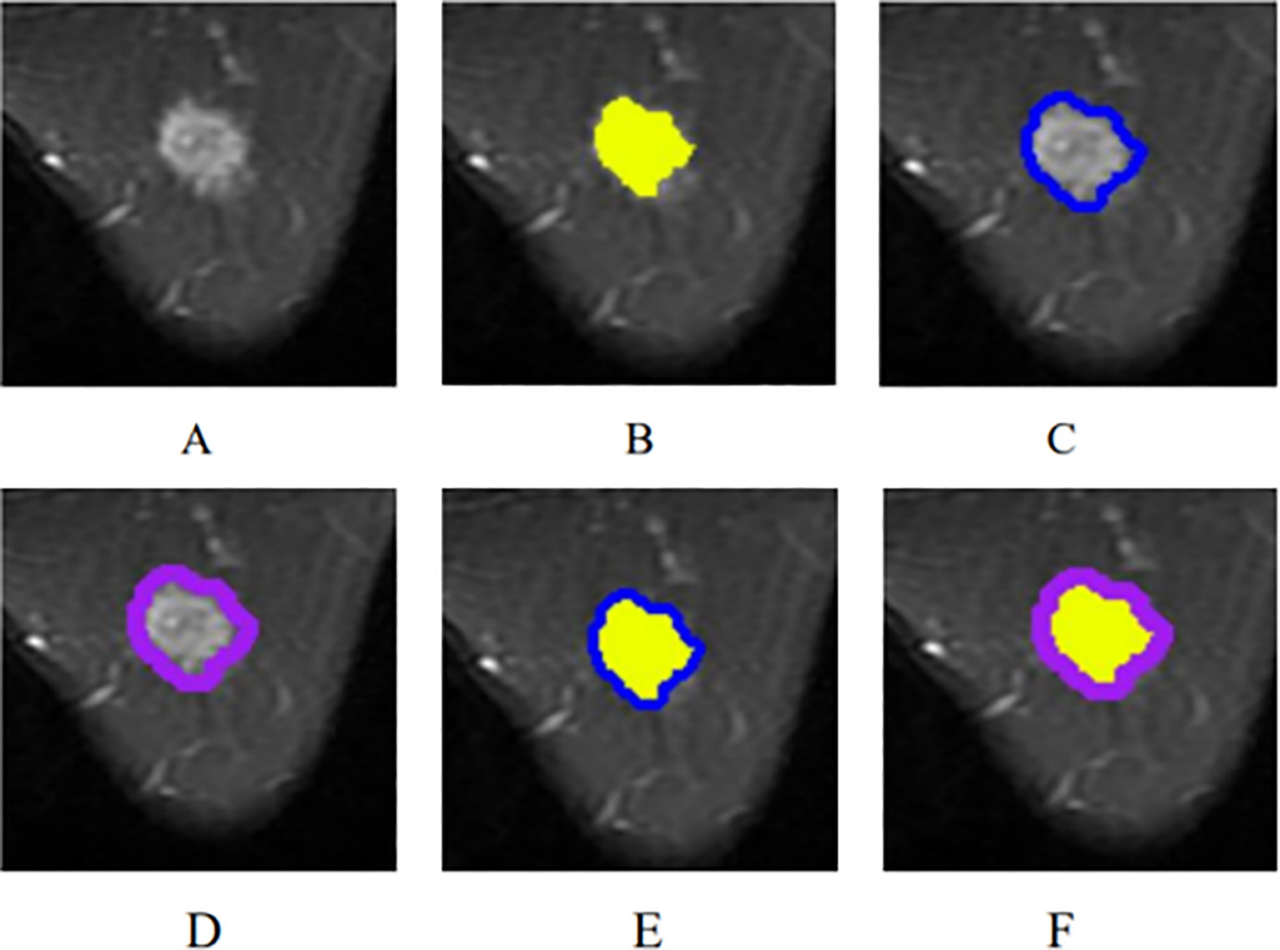
The ROI segmentation of breast cancer lesion (yellow: ITR; blue: PTR-3 mm; purple: PTR-5 mm). **(A)** The original breast mass; **(B)** ITR; **(C)** PTR-3 mm; **(D)** PTR-5 mm; **(E)** ITR+PTR-3 mm; **(F)** ITR+PTR-5 mm.

From the picture archiving and communication systems (PACS), images of axial fat-suppressed T2-weighted sequences were extracted and then saved in DICOM format. By utilizing ITK-SNAP software (Version 3.8.0, http://www.itk-snap.org), the intratumoral region (ITR) was manually delineated slice-by-slice by a breast radiologist with over 5 years of experience who was blinded to the clinicopathological information of the patients, while avoiding the areas of cystic necrosis or hemorrhage. If there were multiple lesions on MRI, the ROI of the largest lesion was delineated. Validation of the delineated ITRs was performed by an associate chief physician with over 10 years of experience in breast radiology. If there was a discrepancy between the ROIs delineated by the two radiologists, a third radiologist with twenty years of experience performed ROI segmentation again and determined the final ROI. The peritumoral regions (PTRs) were obtained by morphologically dilating the ITR outward by 3 mm and 5 mm using the uRP platform (uAI research portal, https://www.uii-ai.com/en/uai/scientifific-research), a clinical research platform incorporating AI module algorithms (23). Any portions of the PTR extending beyond the breast parenchyma were manually removed. Each lesion obtained 3 primary ROIs: the ITR, a PTR-3 mm, and a PTR-5 mm. Additionally, two new combined ROIs were created by merging ITR with the 3 mm and 5 mm PTRs, respectively, to form ITR+PTR-3 mm and ITR+PTR-5 mm regions. (**Figure 2**).

### 2.5 Radiomics feature extraction and selection

The radiomics features calculated from the original MRI images were extracted from the 5 ROIs: ITR, PTR-3 mm, PTR-5 mm, ITR+PTR-3 mm and ITR+ PTR-5 mm using the built-in package PyRadiomics (https://pyradiomics.readthedocs.io/en/latest/index.html) through the uRP platform, including first-order statistical features, shape features, texture features which mainly included Gray Level Cooccurence Matrix (GLCM), Gray Level Run Length Matrix (GLRLM), Gray Level Size Zone Matrix (GLSZM), and filtered features. To calculate filtered features, the first-order and texture features of the MRI images were obtained by various filtering methods such as mean filtering, Gaussian filtering, logarithmic filtering, and wavelet transform, which are described in the **Supplementary Data Sheet 1**. Finally, for each patient, a total of 2264 features from the fat-suppressed T2-weighted sequence were extracted from intratumoral and peritumoral areas, separately.

All extracted features were analyzed as follows. First, the Mann-Whitney U test was performed to select important features that distinguish between luminal and non-luminal features. Second, Z score normalization, a calculation with a mean of 0 and a standard deviation of 1, was used to reduce feature dimensionality differences. Then, high *p* value features were filtered using K-Best (i.e., F value method, K=10). Finally, the radiomics features with the strongest predictivity with differentiating luminal and non-luminal breast cancer were selected by the least absolute shrinkage and selection operator (LASSO) algorithm. The detailed features are shown in the **Supplementary Data Sheet 1**.

### 2.6 Construction of the radiomics score

The radiomics features with the strongest predictivity with differentiating luminal and non-luminal breast cancer were used to construct radscores from ITR, PTR-3 mm, PTR-5 mm, ITR+PTR-3 mm, and ITR+PTR-5 mm, respectively. These radscores were calculated for each patient by the linear combination of selected features and weighted by the respective LASSO coefficients.

### 2.7 Establishment and validation of the clinical-radiomics nomogram

Furthermore, to improve the prediction performance, we further introduced the clinical factors (such as patient age, menstrual status, tumor location, lesion size, BPE, TIC, MRI-reported ALN status, and histological grade) that were correlated with differentiating luminal and non-luminal breast cancer into our predictive models. All clinical factors and radscores based on ITR, PTR-3 mm, PTR-5 mm, ITR+PTR-3 mm, and ITR+PTR-5 mm were evaluated using univariate logistic regression analysis to identify the independent predictors. Multivariate logistic regression analysis was performed to construct the clinical-radiomics model as the predictive model. A nomogram based on the clinical-radiomics model was created to visualize the results for individualized assessment of differentiating luminal and non-luminal breast cancer.

The performance of the models was assessed by sensitivity, specificity, accuracy, and area under the curve (AUC) of receiver operating characteristic (ROC). The calibration of the models was assessed using calibration curves. Decision curve analysis (DCA) compared the net clinical benefits of all the models across a range of threshold probabilities.

### 2.8 Statistical analysis

Statistical analysis was performed using SPSS software (V.26.0) and R software (V. 4.3.1). For the continuous variables, Kolmogorov-Smirnov and Levene were used for normality and homogeneity of variance tests, and the continuous variables that conformed to the normal distribution were expressed as (*χ*_ ± s), and the independent sample t-test was used for comparison between the two groups. The continuous variables conforming to the skewed distribution are expressed as M (Q1, Q3), and the Mann-Whitney U test was used to compare the two groups. The chi-square test was used for comparisons between categorical variables. The AUCs of different models were compared by the DeLong test. A p < 0.05 was considered statistically significant.

## 3 Results

### 3.1 Clinical findings

A total of female 593 breast cancer patients were enrolled in this study, including 421(71%, 421/593) luminal breast cancer patients and 172 (29%,172/593) non-luminal breast cancer patients. The differences in clinical characteristics between training and test sets are shown in **Table 1**. There were no significant differences in the clinical characteristics between the training and test sets (all p > 0.05). There were significant differences in terms of lesion size, MRI-reported ALN status, and histological grade between luminal and non-luminal breast cancer patients (all p < 0.05), but there were no significant differences in the other characteristics (p > 0.05).

**Table 1:** Comparison of Clinical Characteristics Between Luminal and Non-luminal Breast Cancer Groups in the Training and Test sets.

Characteristics	Training Set (n=474)			Test Set (n=119)		
	luminal (n=337)	non-luminal (n=137)	p	luminal (n=84)	non-luminal (n=35)	p
Age [ year/M(Q1,Q3)]	48.00 (41.50,55.00)	49.00 (41.00,55.00)	**0.615**	49.00 (44.25,55.00)	53.00 (43.00,57.00)	0.611
Menstrual status (%)			0.068			0.236
Premenopausal	203 (60.2%)	70 (51.1%)		46 (54.8%)	15 (42.9%)	
Postmenopausal	134 (39.8%)	67 (48.9%)		38 (45.2%)	20 (57.1%)	
Location (%)			0.254			0.129
Left	172 (51.0%)	62 (45.3%)		40 (47.6%)	22 (62.9%)	
Right	165 (49.0%)	75 (54.7%)		44 (52.4%)	13 (37.1%)	
Lesion size [cm /M(Q1,Q3)]	2.00 (1.50,2.80)	2.50 (1.90,3.15)	**0.002**	1.95 (1.50,2.50)	3.00 (1.80,4.10)	**0.001**
BPE (%)			0.466			0.803
Minimal	43 (12.8%)	22 (16.1%)		12 (14.3%)	6 (17.1%)	
Mild	200 (59.3%)	72 (52.6%)		46 (54.8%)	20 (57.1%)	
Moderate	81 (24.0%)	39 (28.5%)		25 (29.8%)	8 (22.9%)	
Marked	13 (3.9%)	4 (2.9%)		1 (2.9%)	1 (1.2%)	
TIC type (%)			0.198			0.594
I	26 (7.7%)	14 (10.2%)		9 (10.7%)	5 (14.3%)	
II	155 (46.0%)	51 (37.2%)		37 (44.0%)	12 (34.3%)	
III	156 (46.3%)	72 (52.6%)		38 (45.2%)	18 (51.4%)	
MRI-reported ALN status (%)			**0.005**			**0.021**
Positive	137 (40.7%	75 (54.7%)		31 (36.9%)	21 (60.0%)	
Negative	200 (59.3%)	62(45.3%)		53 (63.1%)	14 (40.0%)	
Histological grade (%)						
I	22 (6.5%)	10 (7.3%)	**<0.001**	4 (4.8%)	3 (8.6%)	**<0.001**
II	239 (70.9%)	56 (40.9%)		62 (73.8%)	12 (34.3%)	
III	76 (22.6%)	71 (51.8%)		18 (21.4%)	20 (57.1%)	

BPE, background parenchymal enhancement; TIC, time-signal intensity curve; ALN, axillary lymph node.

The bold values presented indicate statistically significant p-values.

### 3.2 Feature extraction and selection

A total of 2264 radiomics features were extracted from each ROI, including 18 first-order statistical features, 14 shape features, 72 texture features, and 2160 filtered features (i.e., high-order statistical features). Finally, 4, 3, 4, 5, and 2 radiomics features were selected as the optimal features based on ITR, PTR-3mm, PTR-5mm, ITR+PTR-3mm, and ITR+PTR-5mm images, respectively, using the LASSO regression method (**Table 2**).

**Table 2:** Radiomics features for each model.

Model	Classification	Feature name	Coefficient
ITR	Filtered feature	wavelet_glrlm_wavelet-LLH-RunVariance wavelet_ngtdm_wavelet-LLL-Contrast wavelet_glrlm_wavelet-LLL-GrayLevelNonUniformityNormalized wavelet_firstorder_wavelet-LLL-Unifomity	0.1072 0.0471 -0.0270 -0.0412
PTR-3mm	Filtered feature	log_firstorder_log-sigma-4-0-mm-3D-MeanAbsoluteDeviation discretegaussian_fistorder_MeanAbsoluteDeviation curvatureflow_firstoder_Variance	0.0621 0.0501 0.0296
PTR-5mm	Filtered feature	recursiveguassion_firstorder_Maximum curvatureflow_firstorder_MeanAbsoluteDeviation log_firstorder_log-sigma-2-0-mm-3D-Variance discretegaussian_fistorder_Variance	0.0539 0.0461 0.0326 0.0120
ITR+PTR-3mm	Filtered feature	normalize_glrlm_LongRunLowGrayLevelEmphasis wavelet_ngtdm_wavelet-LLL-Contrast wavelet_glcm_wavelet-LLL-Idmn normalize_glrlm_LongRunHighGrayLevelEmphasis wavelet_glcm_wavelet-LLL-Idn	0.1512 0.0571 -0.0047 -0.0084 -0.0739
ITR+PTR-5mm	Filtered feature	normalize_glrlm_LongRunLowGrayLevelEmphasis wavelet_glcm_wavelet-LLL-Idmn	0.1281 -0.0918

### 3.3 Radscore evaluation

The formulas for ITR, PTR-3 mm, PTR-5 mm, ITR+PTR-3 mm and ITR+PTR-5 mm radscore were as follows:


$$\begin{array}{l} \text{ITR}\_\text{radscore}=0.1072\times \text{wavelet}\_\text{glrlm}\_\text{wavelet}\_\text{LLH}\_\text{RunVariance}+0.0471\\ \times \text{wavelet}\_\text{ngtdm}\_\text{wavelet}\_\text{LLL}\_\text{Contrast}-0.0270\times \text{wavelet}\_\text{glrlm}\_\text{wavelet}\_\text{LLL}\_\\ \text{GrayLevelNonUniformityNormalized}-0.0412\times \text{wavelet}\_\text{firstorder}\_\text{wavelet}\_\text{LLL}\_\\ \text{Uniformity}+0.2890 \end{array}$$

$$\begin{array}{l} \text{PTR}-3\text{mm}\_\text{radscore}=0.0621\times \text{log}\_\text{firstorder}\_\text{log}\_\text{sigma}\_4\_0\_\text{mm}\_3\text{D}\_\\ \text{MeanAbsoluteDeviation}+0.0501\times \text{discretegaussian}\_\text{firstorder}\_\text{MeanAbsolute}-\\ \text{Deviation}+0.0296\times \text{curvatureflow}\_\text{firstorder}\_\text{Variance}+0.2890 \end{array}$$

$$\begin{array}{l} \text{PTR}-5\text{mm}\_\text{radscore}=0.0539\times \text{recursivegaussian}\_\text{firstorder}\_\text{Maximum}\\ +0.0461\times \text{curvatureflow}\_\text{firstorder}\_\text{MeanAbsoluteDeviation}+0.0326\times \text{log}\_\\ \text{firstorder}\_\text{log}\_\text{sigma}\_2\_0\_\text{mm}\_3\text{D}\_\text{Variance}+0.0120\times \text{discretegaussian}\_\text{firstorder}\\ \_\text{Variance}+0.2890 \end{array}$$

$$\begin{array}{l} \text{ITR}+\text{PTR}-3\text{mm}\_\text{radscore}=0.1512\times \text{normalize}\_\text{glrlm}\_\text{LongRunLowGray}-\\ \text{LevelEmphasis}+0.0571\times \text{wavelet}\_\text{ngtdm}\_\text{wavelet}\_\text{LLL}\_\text{Contrast}-0.0047\times \text{wavelet}\\ \_\text{glcm}\_\text{wavelet}\_\text{LLL}\_\text{ldmm}-0.0084\times \text{normalize}\_\text{glrlm}\_\text{LongRunHighGrayLevel}-\\ \text{Emphasis}-0.0739\times \text{wavelet}\_\text{glcm}\_\text{wavelet}\_\text{LLL}\_\text{Idn}+0.2890 \end{array}$$

$$\begin{array}{l} \text{ITR}+\text{PTR}-5\text{mm}\_\text{radscore}=0.1281\times \text{normalize}\_\text{glrlm}\_\text{LongRunLow}-\\ \text{GrayLevelEmphasis}-0.0918\times \text{wavelet}\_\text{glcm}\_\text{wavelet}\_\text{LLL}\_\text{ldmm}+0.2890 \end{array}$$

**Figure 3:**
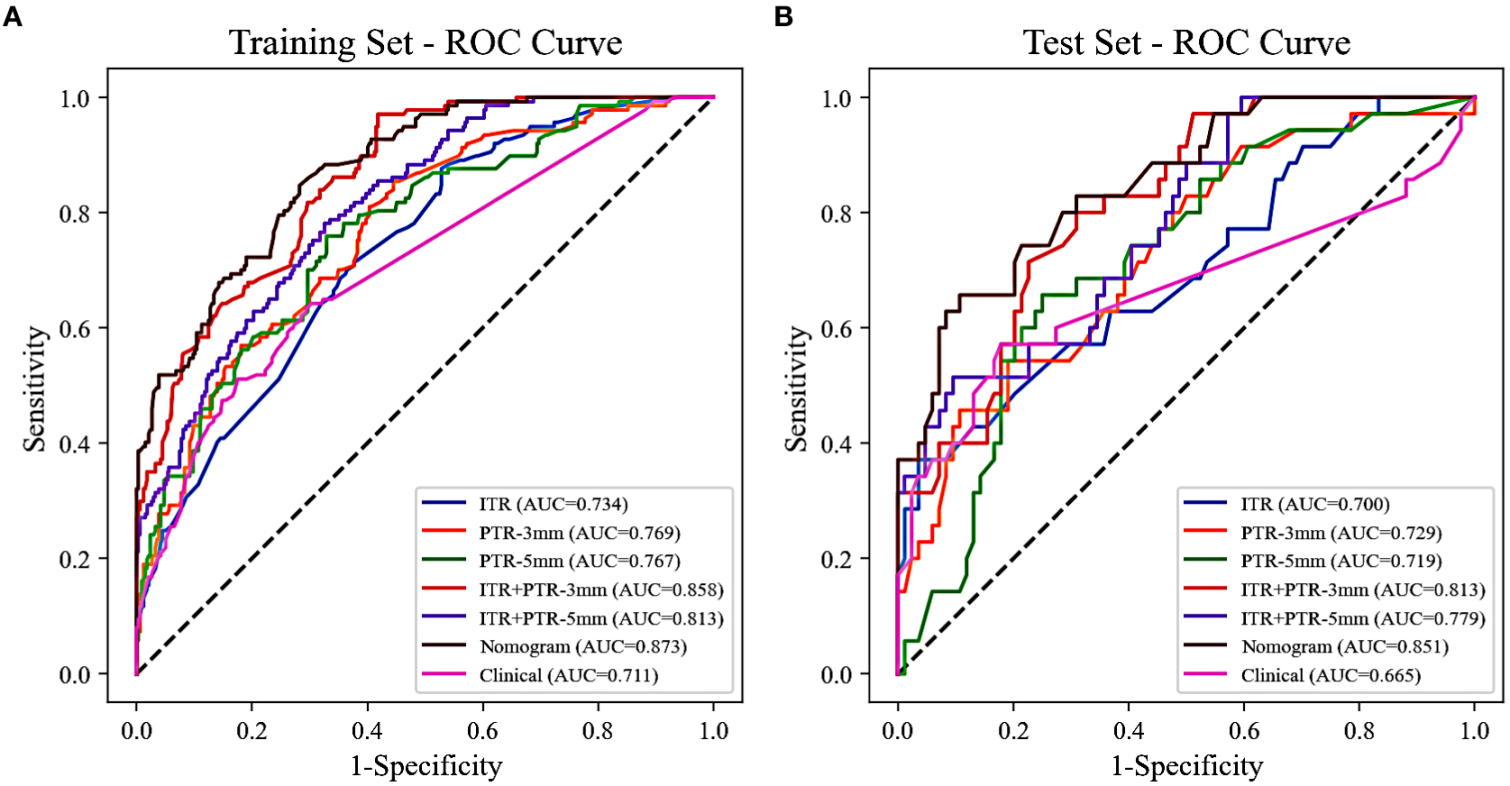
The ROC curves of all the models were used to differentiate luminal and non-luminal breast cancer in the training **(A)** and test **(B)** sets. ROC, receiver operating characteristic; AUC, the area under the curve of ROC.

The AUCs for ITR, PTR-3mm, PTR-5mm, ITR+PTR-3mm, and ITR+PTR-5mm radscore were 0.734, 0.769, 0.767, 0.858, 0.813 in the training set and 0.700, 0.729, 0.719, 0.813, 0.779 in the test set, respectively (**Figure 3**).

### 3.4 Construction and evaluation of the clinical-radiomics nomogram

**Figure 4:**
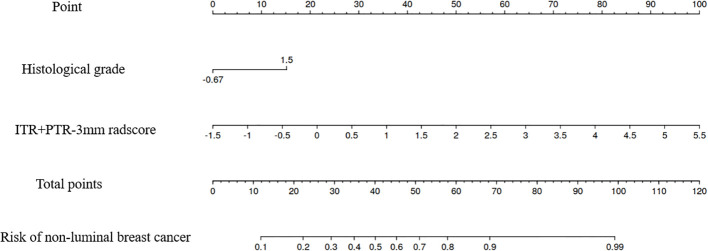
The nomogram of the model based on the ITR+PTR-3mm radscore and clinical characteristic. The radiomics nomogram integrated the ITR+PTR-3mm radscore with the histological grade in the training cohort.

**Figure 5:**
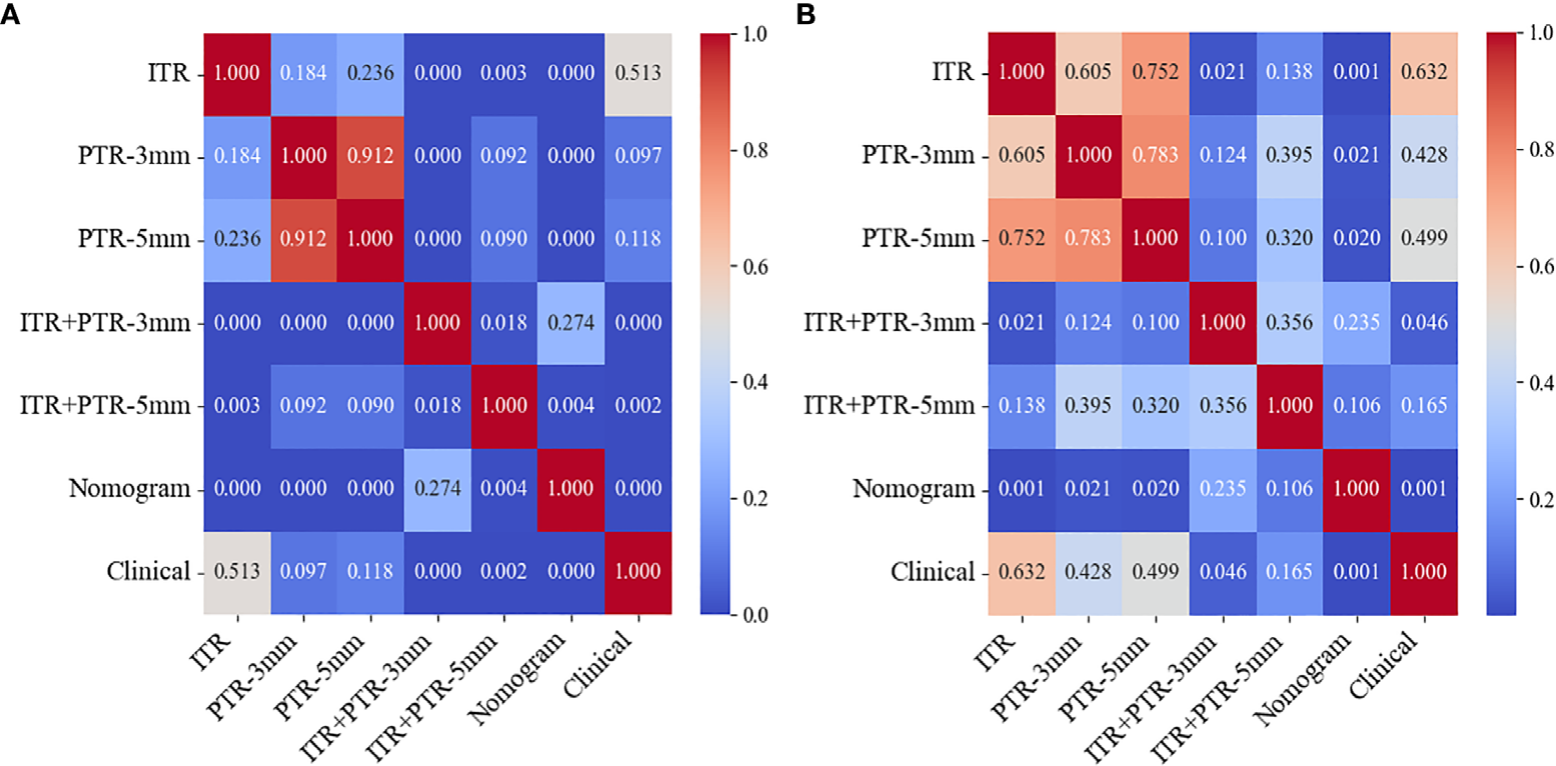
Heatmaps of Delong test between any two models in the training **(A)** and test **(B)** sets.

**Figure 6:**
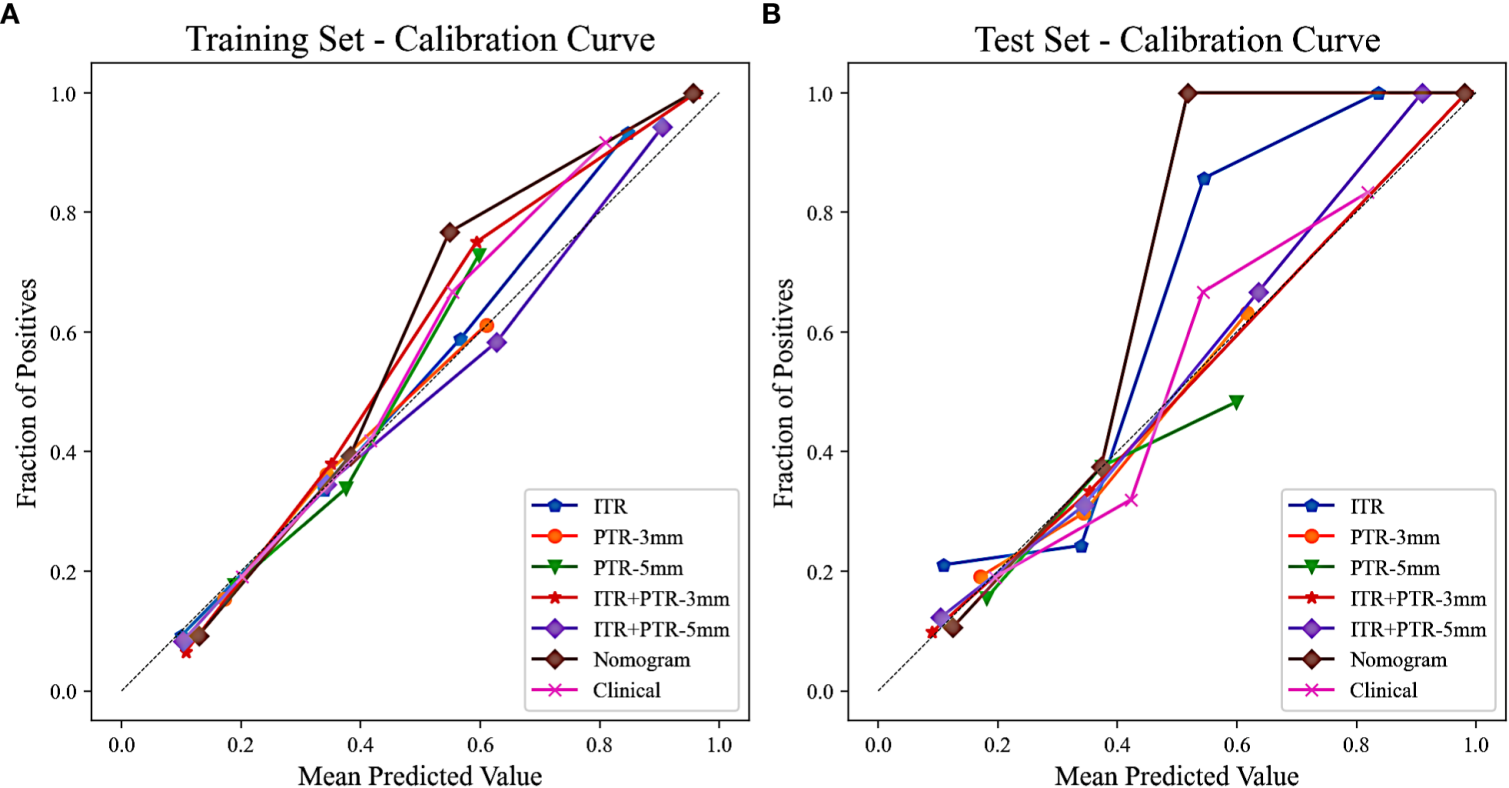
Calibration curves of all the models in the training **(A)** and test **(B)** sets. The calibration curves show the agreement between the predicted probability of differentiating luminal and non-luminal breast cancer and the actual outcomes.

**Figure 7:**
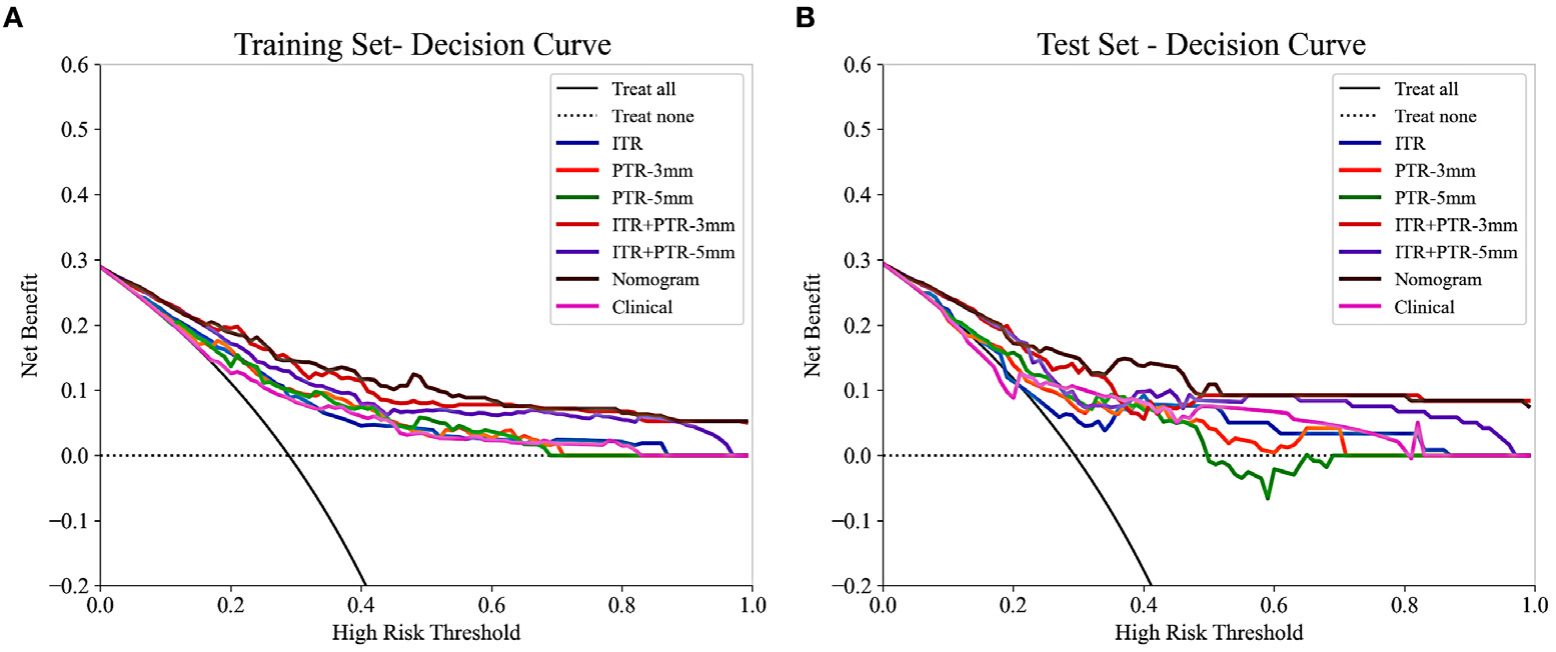
The decision curves of all the models in the training **(A)** and test **(B)** sets, with threshold probability on the x-axis and net benefit on the y-axis. The nomogram showed the highest net benefit within the threshold probability range of 0.01-0.99, with the widest applicable range.

Based on the univariate and multivariate logistic regression analyses, two risk factors (ITR+PTR-3mm radscore and histological grade) were obtained for differentiating luminal and non-luminal breast cancer (**Table 3**). A clinical model was constructed by histological grade. A clinical-radiomics model was constructed by adding ITR+PTR-3mm radscore to the clinical factor. The nomogram is constructed for visualizing the clinical-radiomics model (**Figure 4**). The sensitivity, specificity, accuracy, and AUC for the nomogram were 72.3%,78.9%,77.0% and 0.873 in the training set and 71.4%, 79.8%, 77.3%, and 0.851 in the test set (**Table 4**). The DeLong test showed that the AUCs of the ITR, PTR-3mm, PTR-5mm, and clinical model were significantly different from that of the nomogram in the test set (p = 0.001, p=0.021, p=0.020, and p = 0.001, respectively), and the results are shown in **Figure 5**. The calibration curves indicated that there was good agreement between the predicted risk and the observed probability across the whole dataset (**Figure 6**). The decision curves showed that the nomogram had the best clinical net benefit across threshold probabilities of 0.01-0.99 and the widest applicable range compared to other models (**Figure 7**).

**Table 3:** Logistic Regression Analysis for Clinical Characteristics.

Parameters	Univariate analysis		p value	Multivariate analysis		p value
	OR	95%CI		OR	95%CI	
ITR radscore	1.646	1.438,1.884	<0.001*	NA	NA	NA
PTR-3mm radscore	1.674	1.422,1.971	<0.001*	NA	NA	NA
PTR-5mm radscore	1.656	1.407, 1.949	<0.001*	NA	NA	NA
ITR+ PTR-3mm radscore	3.694	2.424, 5.631	<0.001*	3.163	1.959, 5.107	**<0.001**
ITR PTR-5mm radscore	1.758	1.542, 2.006	<0.001*	NA	NA	NA
Age	1.000	0.998, 1.002	0.614	NA	NA	NA
Menstrual status	0.689	0.462, 1.029	0.068	NA	NA	NA
Location	0.793	0.532, 1.181	0.254	NA	NA	NA
Lesion size	1.002	1.000, 1.004	0.002*	NA	NA	NA
BPE	0.704	0.394, 1.257	0.235	NA	NA	NA
TIC Curve	0.611	0.297, 1.259	0.182	NA	NA	NA
MRI-reported ALN status	1.744	1.198, 2.669	0.004*	NA	NA	NA
Histological grade	2.138	1.790, 2.555	<0.001*	1.785	1.392, 2.289	**<0.001**

OR, odds ratio; CI, confidence interval; BPE, background parenchymal enhancement; TIC, time-signal intensity curve; ALN, axillary lymph node.

The bold values presented indicate statistically significant p-values.

**Table 4:** Predictive Performances Among Different Models.

Models	Training Set (n=560)				Test Set (n=140)			
	AUC (95% CI)	SEN	SPE	ACC	AUC (95% CI)	SEN	SPE	ACC
ITR	0.734 (0.687-0.781)	68.6%	64.7%	65.8%	0.700 (0.593-0.807)	62.9%	65.5%	64.7%
PTR-3mm	0.769 (0.723-0.815)	71.5%	62.3%	65.0%	0.729 (0.629-0.829)	65.7%	61.9%	63.0%
PTR-5mm	0.767 (0.721-0.814)	61.3%	74.2%	70.5%	0.719 (0.621-0.817)	71.4%	60.7%	63.9%
ITR+PTR-3mm	0.858 (0.824-0.891)	72.3%	73.0%	72.8%	0.813 (0.735-0.891)	77.1%	69.0%	71.4%
ITR+PTR-5mm	0.813 (0.774-0.853)	77.4%	67.4%	70.3%	0.779 (0.690-0.867)	68.6%	64.3%	65.5%
Nomogram	0.873 (0.841-0.906)	72.3%	78.9%	77.0%	0.851 (0.778-0.925)	71.4%	79.8%	77.3%
Clinical	0.711 (0.661-0.762)	64.2%	69.7%	68.1%	0.665 (0.542-0.789)	60.0%	72.6%	68.9%

AUC, area under the receiver operating characteristic curve; CI, confidence interval; SEN, sensitivity; SPE, specificity; ACC, accuracy.

## 4 Discussion

In this study, intratumoral and peritumoral radiomics models based on the fat-suppressed T2 sequence were developed that are capable of accurately differentiating luminal and non-luminal breast cancer. Furthermore, a nomogram constructed by combining intratumoral and peritumoral radscore and clinical characteristics was clinically useful in helping therapeutic strategy optimization.

In the non-luminal group, the percentages of MRI reported-positive ALN (Training set: 54.7% > 40.7%; Test set: 60.0% > 36.9%) and histological grade III (Training set: 51.8% > 22.6%; Test set: 57.1% > 21.4%) were higher than those in the luminal group, lesion size was also larger in the non-luminal group (Training set: 2.50>2.00; Test set: 3.00>1.95), and all the differences were statistically significant (all p < 0.05). In addition, ITR+PTR-3mm radscore and histological grade were determined as the risk factors by using univariate and multivariate logistic regression (p < 0.05). This is consistent with Feng’s study for choosing histological grade as a risk factor (24), probably due to histological grade as one of the prognostic indicators of breast cancer (25), with higher grades, indicating a worse prognosis, so the grades of non-luminal breast cancer tend to be higher than that of luminal breast cancer. Furthermore, in addition to histological grade, our study also found ITR+PTR-3mm radscore as a risk factor, which may be related to the fact that the ITR+PTR-3mm radscore may be indicative of a more extensive tumor environment, potentially correlating with a higher risk of recurrence or poorer response to standard therapies. However, this is different from Huang’s study (26), which screened for no risk factors, which may be due to differences in the subjects and MRI sequences, which only focused on intratumoral radiomics regions and selected DCE-MRI sequences. Therefore, ITR+PTR-3mm radscore and histological grade could be used as the predictors in our model.

Radiomics methods have been reported to preoperatively differentiate luminal and non-luminal breast cancer in certain malignant tumors (24, 26). However, further study on the value of fat-suppressed T2 sequence for preoperatively differentiating luminal and non-luminal breast cancer is still needed. In our study, the AUCs for ITR, PTR-3mm, PTR-5mm, ITR+PTR-3mm, and ITR+PTR-5mm radscores based on the fat-suppressed T2-weighted images were 0.734, 0.769, 0.767, 0.858 and 0.813 in the training set and 0.700, 0.729, 0.719, 0.813 and 0.779 in the test set, respectively. Our intratumoral+peritumoral model is comparable to that of Huang et al. (0.813、0.779 VS 0.80) (26), and the AUC of our intratumoral or peritumoral model alone was slightly lower than its AUC (0.700、0.729、0.719<0.80), which utilized DCE-MRI-based radiomics features to distinguish between luminal and non-luminal breast cancers. Feng et al. used a nomogram combining clinical factors with a radiomics score based on the DCE-MRI features of the intratumoral subregion to distinguish between luminal and non-luminal breast cancers (24), with an AUC of 0.830 in the training set and 0.879 in the test set, which were comparable to our study (training set:0.873; test set:0.851). Although different imaging sequences were used. In our study, the fat-suppressed T2 sequence was employed to emphasize differences in tissue contrast, which may capture additional aspects of tumor heterogeneity not as evident in DCE-MRI. The comparable performance of our nomogram suggests that T2-weighted imaging can be as effective as DCE-MRI when combined with radiomics analysis, possibly due to the unique tissue characteristics highlighted in this sequence. This underscores the potential of using alternative MRI sequences in clinical-radiomics models to improve diagnostic accuracy and adapt to specific clinical needs, enhancing personalized treatment planning for breast cancer patients.

The peritumor microenvironment can also reflect the biological characteristics of the tumor to a certain extent (27). Previous studies have demonstrated the strong predictive effect of peritumoral radiomics features in the diagnosis of breast cancer, lymph node metastasis status, molecular typing, neoadjuvant chemotherapy efficacy, HER2 expression status, KI-67 expression level, lymphovascular invasion and programmed cell death ligand 1 (PD-L1) expression status (28–35). Our study found that the AUCs of both the PTR-3 mm and PTR-5 mm radiomics models were higher than that of the ITR model, with the PTR-3 mm model exhibiting a higher AUC than the PTR-5 mm model. This may be related to the smaller peritumoral radius increasing the sensitivity to the lesion, thereby improving the predictive performance of the model, which is consistent with the findings of Park et al. on the value of predicting neoadjuvant chemotherapy (NAC) pathological complete response (31), they demonstrated that the predictive performance of the PTR-1 mm radiomics model was higher than that of the PTR-3 mm radiomics model, regardless of the early stage of the enhanced scan (AUC:0.940>0.900) or the late stage of the enhanced scan (AUC:0.890>0.720), or the T2WI sequence (AUC:0.920>0.870).

Furthermore, when combined with intratumoral region, the AUC of PTR-3 mm reached 0.858 (training set) and 0.813 (test set) and the AUC of PTR-5 mm reached 0.813 (training set) and 0.779 (test set) in our study. In addition, the ITR+ PTR-3 mm and ITR+ PTR-5 mm models showed improved sensitivity, specificity, and accuracy compared to the ITR model. Besides, the AUC of the ITR+PTR-3 mm model was higher than that of the ITR+PTR-5 mm model. This may be related to the fact that the ITR+PTR-3 mm model captures a more optimal balance of relevant tumor characteristics by focusing on a narrower peritumoral margin, which can more accurately reflect the invasive properties of the tumor. In contrast, the ITR+PTR-5 mm model, by incorporating a wider margin, may include more non-tumor tissue, potentially diluting the precision of the radiomic features. This suggests that a tighter peritumoral margin in radiomics analysis may yield better diagnostic performance by focusing on more critical tumor-related changes.

The nomogram was established by incorporating radscore with ITR+PTR-3 mm and histological grade in our study, which also was a visual representation of the combined model. The nomogram in our study showed higher predictive efficacy than that of independent method of radiomics radscore and clinical feature model, and the sensitivity, specificity, accuracy, and AUC for the nomogram were 72.3%, 78.9%, 77.0%, and 0.873 (95% CI: 0.841-0.906) in the training set and 71.4%, 79.8%, 77.3%, and 0.851 (95% CI:0.778-0.925) in the test set, respectively. However, although the nomogram exhibited higher AUC values compared to the ITR+PTR-3mm and ITR+PTR-5mm models (AUC: 0.851 VS 0.813、0.779), the differences were not statistically significant (p = 0.235、0.106). This may be attributed to the limited number of risk factors included, which, although significant, may not capture enough variability to demonstrate clear superiority over simpler models in the test set. Additionally, the sample size and potential overfitting could have affected the statistical power needed to discern subtle improvements. Future studies may benefit from incorporating a broader set of risk factors and employing larger, more diverse datasets to enhance model robustness and detect statistically significant differences in performance. DCA also showed that the nomogram had the best clinical net benefit and widest applicable range compared to the other models, which indicates that the nomogram has promising clinical application value.

There were some limitations in our study. First, the ROI outline of breast cancer lesions is manually delineated by radiologists, which is time-consuming, subjective, error-prone, and not scalable with increasing data volumes. Therefore, an automated, standardized, repeatable, and validated segmentation method would be more practical for future use. Second, as a single-center retrospective study, there may be selection bias. The results need external validation, which would be conducted using datasets from multiple institutions in the subsequent studies. Third, the use of a single 3T GE MR scanner introduces limitations due to potential differences in scanner hardware and imaging protocols among different vendors and field strengths, such as 1.5T scanners. These discrepancies might impact the reproducibility of our radiomics features. To enhance the robustness and general applicability of our models, future research should investigate these findings using a variety of scanner types across multiple clinical settings. Finally, the peritumoral region was obtained by dilating the tumor 3 mm and 5 mm in this study, but whether this is the optimal peritumoral region requires further refinement of the expanded range and validation.

## 5 Conclusion

In conclusion, our study suggests that intratumoral and peritumoral radiomics methods based on the fat-suppressed T2 sequence are helpful for preoperative accurately differentiating luminal and non-luminal breast cancer, especially the clinical-radiomics nomogram could provide a new strategy for personalized treatment of breast cancer patients in clinical practice.

## Data Availability

The data analyzed in this study is subject to the following licenses/restrictions: The raw data supporting the conclusions of this article will be made available by the authors, without undue reservation. Requests to access these datasets should be directed to Yaxin Guo, gyaxinnn@126.com.
