# Supplementary material for: Clinical-radiomics nomogram based on the fat-suppressed T2 sequence for differentiating luminal and non-luminal breast cancer
Source: Front Oncol. 2024 Oct 25;14:1451414. doi: 10.3389/fonc.2024.1451414 (PMC11543577; doi:10.3389/fonc.2024.1451414)
Supplement: Supplementary file 1 [file DataSheet1.docx]

**Supplementary Data**

***Radiomics feature extraction****:*

A total of 2264 radiomics features were extracted from each ROI, including 18 first-order statistical features, 14 shape features, 72 texture features and 2160 filtered features (i.e., high-order statistical features).

Part I: Shape Features

The shape feature describes the spatial geometry of the tumor, and there are 8 shape features in our study. The description of shape features is shown in Table S1.

**Table S1: Shape Features**

| Index | Feature Name | Explanation |
| --- | --- | --- |
| 1 | Volume | Target tumor volume |
| 2 | Surface-to-volume ratio | The ratio of surface area to volume of target tumor |
| 3 | Sphericity | Measure the similarity between ROI and sphere |
| 4 | Compactness1 | A quantitative description of the compactness of tumor |
| 5 | Compactness2 | A quantitative description of the compactness of tumor |
| 6 | Spherical disproportion | Reflecting the asymmetry of tumor |
| 7 | Maximum 3D diameter | Maximum 3-D diameter and length of tumor |
| 8 | Eccentricity | Reflecting the ellipsoidal feature that best matches the area of the tumor |

Part II: First-order Statistical features

The first-order statistical feature is extracted from the histogram, including 17 statistical features, and the description of first-order statistical features is shown in Table S2.

**Table S2: First-order Statistical Features**

| Index | Feature Name | Explanation |
| --- | --- | --- |
| 1 | Energy | The sum of the squares of the gray values corresponding to all the voxels in the ROI reflected the change in the gray value. |
| 2 | Entropy | Describe the uncertainty of the source, which is a measure of the degree of system ordering. |
| 3 | Minimum | Minimum value of voxel gray value |
| 4 | Maximum | Maximum gray value of voxel |
| 5 | Mean | Mean value of voxel gray value |
| 6 | Median | The value of the middle position after the gray value sorted |
| 7 | Interquartile Range | Difference between 3/4 and 1/4 quantiles of brightness in ROI |
| 8 | Range | The difference between the maximum and minimum gray values |
| 9 | Mean absolute Deviation | The sum of the distance between the observed value of each voxel gray value and the average value. |
| 10 | Robust Mean Absolute Deviation | Strong mean absolute deviation, the average distance of all intensity values calculated on a subset of image arrays, where the gray level is in or equal to 10-digit and 90-digit |
| 11 | Root mean square | The sum of squares of voxel gray values is divided by the number of voxels |
| 12 | Standard deviation | Standard deviation reflects the discreteness of data |
| 13 | Skewness | Standard deviation reflects the discreteness of data. The measurement of the skewed direction and degree of statistical data distribution is a digital feature of the asymmetric degree of statistical data distribution. Intuitively, it is the relative length of the tail of the density function curve |
| 14 | Kurtosis | The characteristic number represents the peak value of the probability density distribution curve at the average value. Intuitively, the kurtosis reflects the thickness of the tail. |
| 15 | Variance | Measuring the dispersion of data |
| 16 | Uniformity | The discreteness of the measured data reflects the uniformity of the percentage of gray value histogram statistics. The bigger the value is, the more uniform the value is, the smaller the value is, the more complex it is. |
| 17 | Peak | The maximum of the mean value of 26 voxels linked to each other using the tumor voxels as nuclei. |

Part III: Texture Features

Texture features are used to describe the similarity between voxels. A total of 54 texture features are used in this project, including 22 GLCM (Gray-level co-occurrence matrix), 14 GLRLM (Gray-level run-length matrix) features, 13 GLSZM (Gray-level size zone matrix) features and 5 NGTDM (Neighboring gray-tone difference matrix) features. The description of texture features is shown in Table S3.

**Table S3: Texture Features**

| Category | Feature Name | Explanation |
| --- | --- | --- |
| GLCM | Autocorrelation | The autocorrelation coefficient of the co-occurrence matrix |
|  | Cluster prominence | Outburst clustering |
|  | Cluster shade | Dark clustering |
|  | Cluster tendency | Trend clustering |
|  | Contrast | Reflecting the clarity of the image and the depth of the texture trench |
|  | Correlation | The measurement of linear dependence of gray level in image |
|  | Difference entropy | Difference entropy |
|  | Dissimilarity | The difference variance measure |
|  | Joint energy | Measurement of image uniformity |
|  | Joint entropy | Measurement of random/variability of neighborhood strength value |
|  | Homogeneity1 | Homogeneity of ROI |
|  | Homogeneity2 | Homogeneity of ROI |
|  | Informational measure of correlation1 1 | Mutual Information Measure 1 |
|  | Informational measure of correlation1 2 | Mutual information measure 2 |
|  | Inverse difference moment normalized | Normalized inverse moment |
|  | Inverse difference normalized | The significance of reciprocity and inertia is just the opposite. |
|  | Inverse variance | Inverse variance |
|  | Maximum probability | The maximum probability of the occurrence of adjacent strength values |
|  | Sum Average | Gray mean probability sum |
|  | Inverse difference | Inverse difference |
|  | Sum of Squares | A measure of the stability of gray level change of image texture, reflecting the uniformity of gray level distribution and texture roughness |
|  | Sum Entropy | A sum of neighborhood intensity value differences |
| GLRLM | Short Run Emphasis | Short-range focus |
|  | Long Run Emphasis | Key points of long journey |
|  | Gray Level Non-uniformity | Gray level inhomogeneity |
|  | Run-Length Non-uniformity | Inhomogeneity of travel length |
|  | Run-Length Non-uniformity normalized | Normalized stroke length nonuniformity |
|  | Run Percentage | Percentage of travel |
|  | Run variance | Travel variance |
|  | Run Entropy | Entropy of travel |
|  | Low Gray-Level Run Emphasis | Key operating points of low gray level |
|  | High Gray-Level Run Emphasis | Key operating points of high gray level |
|  | Short Run Low Gray-Level Emphasis | Key points of low gray level for short travel |
|  | Short Run High Gray-Level Emphasis | Key points of short range and high gray level |
|  | Long Run Low Gray-Level Emphasis | Key points of long travel and low gray level |
|  | Long Run High Gray-Level Emphasis | Key points of long travel and high gray level |
| GLSZM | Small Area Emphasis | Small area focus |
|  | Large Area Emphasis | Large area focus |
|  | Gray-Level Non-uniformity | Gray level inhomogeneity |
|  | Gray-Level Non-uniformity Normalized | Normalized gray level inhomogeneity |
|  | Zone-Size Non-uniformity Normalized | Size inhomogeneity of normalized region |
|  | Zone Percentage | Regional ratio |
|  | Gray Level Variance | Gray variance |
|  | Zone Variance | Regional variance |
|  | Low Gray Level Zone Emphasis | Low gray level region focus |
|  | High Gray Level Zone Emphasis | Highly gray level region focus |
|  | Small Area Low Gray Level Emphasis | Key points of small area low gray level |
|  | Small Area High Gray Level Emphasis | Key points of small area and high gray level |
|  | Large Area Low Gray Level Emphasis | Key points of large area low gray level |
| NGTDM | Coarseness | Measurement of the mean difference between central voxels and their neighbors |
|  | Contrast | Measurement of spatial intensity change |
|  | Busyness | Measuring the change from pixel to its neighborhood |
|  | Complexity | Image complexity measurement |
|  | Strength | Measurement of primitives in images |

Part IV: Filtered Features

Filtered features consist of 432 gray statistical features and 1728 texture features extracted from the filtered image, and 24 filtering methods include mean filtering, Gaussian filtering, logarithmic filtering, and wavelet transform. The description of filtered features is shown in Table S4.

**Table S4: Filtered Features**

| Index | Filter | Explanation |
| --- | --- | --- |
| 1 | Box mean | Box-averaging filter, which uses the accumulator method to implement a fast rectangular mean filter |
| 2 | Additive Gaussian  noise | Additive Gaussian noise filter to change the image with additive Gaussian white noise |
| 3 | Binomial blur image | Item-smoothed filter that performs separable blur on each dimension of the image |
| 4 | Curvature flow | Curvature Flow Filter: Denoise an image using a curvature-driven stream |
| 5 | Box sigma image | Box filter, fast rectangular sigma filter using the accumulator method (different from the first parameter) |
| 6 | LoG | The Laplacian of Gaussian filter is an edge-enhancing filter. To use it, you need to specify the parameter sigma, with low sigma emphasizing fine textures and high sigma values emphasizing coarse sticky textures |
| 7 | Wavelet | Wavelet transform. Generate all combinations (LLH, LHL, LHH, HUHLHL, HHL, HH, TLL) using high-pass and low-pass filters for each of the three dimensions |
| 8 | Normalize | A normalization filter that normalizes an image by setting the mean value of the image to zero and the variance to one |
| 9 | Laplacian sharpening | Laplace sharpening filter, which sharpens the image with the Laplace operator |
| 10 | Discrete Gaussian | Discrete Gaussian filter, a separable convolutional blurred image through discrete Gaussian kernels |
| 11 | Mean | Mean filter |
| 12 | Speckle noise | Speckle noise filter |
| 13 | Recursive Gaussian | A recursive Gaussian filter that approximates the basis class of IIR convolution with a Gaussian kernel |
| 14 | Shot noise | Shot noise filter |

***Radiomics feature selection***

To eliminate the difference in the range of feature values, the Mann-Whitney U test was performed before feature selection. Z score normalization, K best method, and LASSO regression methods were used to select the radiomics features with the strongest predictivity with differentiating luminal and non-luminal breast cancer. Each 4, 3, 4, 5, and 2 radiomics features were finally selected as the optimal features based on ITR, PTR-3mm, PTR-5mm, ITR+PTR-3mm, and ITR+PTR-5mm images using the LASSO regression method, respectively. The detailed radiomics features for each model are illustrated in the **Figure S1**.

**Figure S1:**


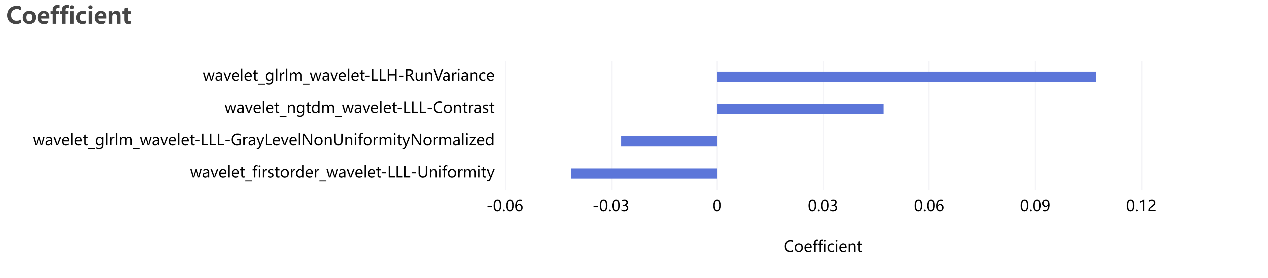


**(1) 4 radiomics features based on ITR.**


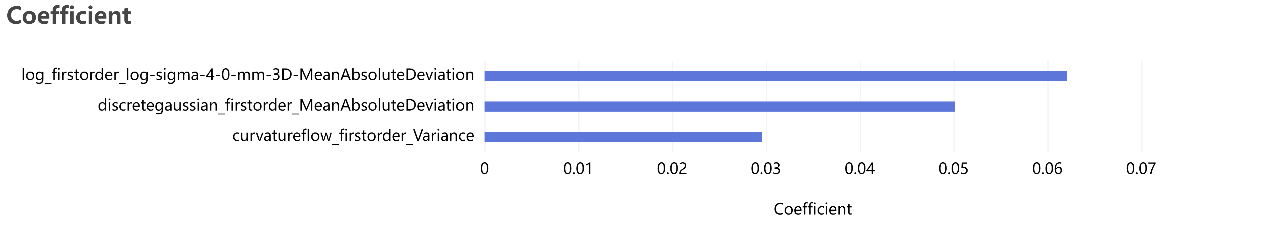


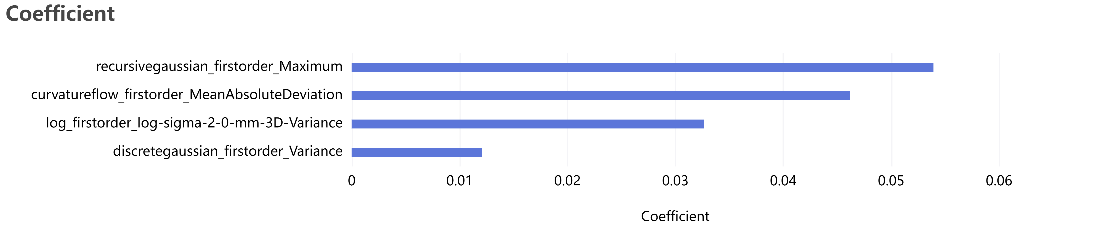
**(2) 3 radiomics features based on PTR-3mm.**

**(3) 4 radiomics features based on PTR-5mm.**


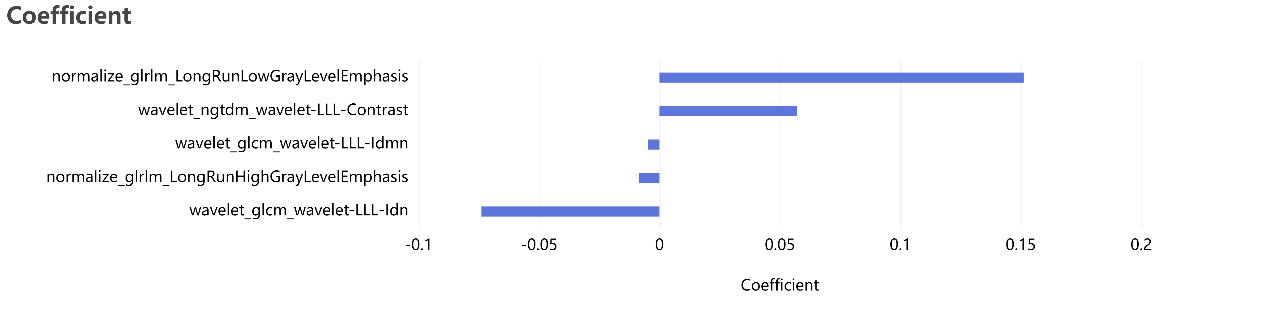


**(4) 5 radiomics features based on ITR+PTR-3mm.**


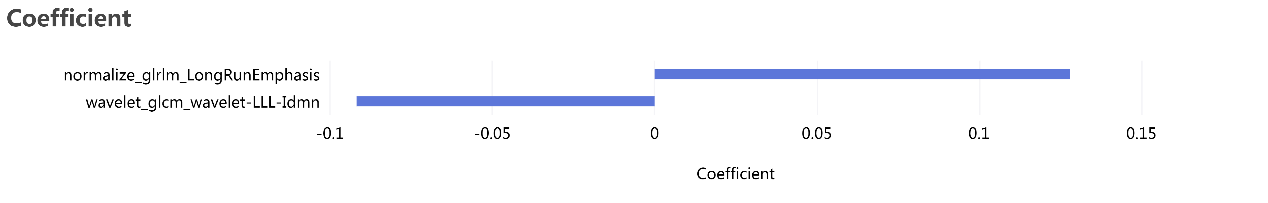


**(5) 2 radiomics features based on ITR+PTR-5mm.**
